# Manganese methionine hydroxy analog chelated affects growth performance, trace element deposition and expression of related transporters of broilers

**DOI:** 10.1016/j.aninu.2020.09.005

**Published:** 2021-03-11

**Authors:** Tiantian Meng, Lumin Gao, Chunyan Xie, Yangkui Xiang, Yiqiang Huang, Yawei Zhang, Xin Wu

**Affiliations:** a CAS Key Laboratory of Agro-ecological Processes in Subtropical Region, Institute of Subtropical Agriculture; National Engineering Laboratory for Pollution Control and Waste Utilization in Livestock and Poultry Production; Hunan Provincial Engineering Research Center for Healthy Livestock and Poultry Production, Changsha, 410125, China; b Hunan Co-Innovation Center of Safety Animal Production, College of Animal Science and Technology; College of Resources and Environment, Hunan Agricultural University, Changsha, 410128, China; c Tianjin Institute of Industrial Biotechnology, Chinese Academy of Sciences, Tianjin, 300308, China; d Hunan Provincial Research Center of Mineral Element Nutrition Engineering Technology, Xing-Jia Bio-engineering Co., Ltd., 410300, Changsha, China

**Keywords:** Manganese methionine hydroxy analog chelated, Trace element, Deposition, Transporter, Broiler

## Abstract

The present study aimed to evaluate the effects of manganese methionine hydroxyl analog chelated (Mn-MHAC) as a manganese (Mn) source on growth performance and trace element deposition in broilers. A total of 432 Arbor Acres commercial female broilers were fed a basal corn-soybean diet containing Mn at 25.64 mg/kg diet for 10 d. They were then randomly assigned to 6 groups, including a control group (the basal diet), a Mn sulfate group (the basal diet supplemented with Mn at 100 mg/kg diet), and 4 Mn-MHAC groups (the basal diet supplemented with Mn-MHAC at 25, 50, 75 and 100 mg Mn/kg diet, respectively). The results showed that compared with the control group, groups supplemented with Mn-MHAC had a positive effect on BW (quadratic*, P* = 0.017) and ADG (quadratic, *P* = 0.017). Moreover, the Mn-MHAC (50 mg Mn/kg diet) group had significantly greater BW and ADG (*P* < 0.05) compared with the other Mn-MHAC groups. Trace element deposition results also showed that tibial Mn increased (linear or quadratic, *P* = 0.002 and 0.009, respectively) in groups fed diets with increased levels of Mn-MHAC. In contrast, Fe deposition decreased both in the heart (linear, *P* = 0.020) and tibia (*P* < 0.05). In addition, the Mn-MHAC supplement noticeably lowered serum Mn-SOD activity (linear or quadratic, *P* = 0.048 and 0.019, respectively). The relative mRNA levels of divalent metal transporter 1 (*DMT1*) (*P* = 0.024), ferroportin 1 (*FPN1*) (*P* = 0.049), and Cu transporter-1(*CTR1*) (*P* < 0.001) in the duodenum, as well as *CTR1* in the jejunum and ileum (*P* = 0.040 and 0.011, respectively) were higher in the Mn-supplemented group than in the control group. Furthermore, the relative mRNA level of *DMT1* in the jejunum and ileum of broilers in the Mn-MHAC group (50 mg Mn/kg diet) did not differ from those in the control group, but was lower than those in the Mn sulfate group (*P* < 0.05). In conclusion, Mn-MHAC dietary supplementation improved the growth performance and trace element deposition in broilers. From this study, we recommend the optimum Mn-MHAC level to meet the Mn requirement of broilers is 50 to 75 mg Mn/kg diet.

## 1 Introduction

As an essential trace element, manganese (Mn) plays an important role in various biological activities, including as an essential cofactor for superoxide dismutase, transferases, hydrolases, and lyases (Bagga and Patel, 2012; Soetan, 2010). Mn deficiency, particularly in the growing chick, is closely associated with multiple adverse outcomes, including poor growth, decreased bone Mn concentrations, and skeletal abnormalities. However, overexposure of Mn also disturbs the homeostasis of trace elements, including iron (Fe) and copper (Cu) in the tissues (Bai et al., 2014; Bao et al., 2010). Thus, optimal Mn utilization is an increasing concern for the extremely rapid growth rate of commercial broiler strains. Inorganic Mn supplements are routinely added to conventional poultry diets to meet Mn requirements. However, the absorption of minerals in inorganic form by broilers is limited, primarily due to their tendency to form complexes with other dietary constituents that are less available or unavailable, and their interference with each other in the digestive tract (Wan et al., 2018). It is generally accepted that the organic Mn complex or chelates have better absorption or bio-efficacy and less environmental contamination than inorganic sources (Ji et al., 2006; Xiao et al., 2014). However, previous studies about the effects of different organic Mn complex or chelates supplementation on the growth performance of broiler are inconsistent. For instance, the Mn fumarate (Berta et al., 2004) and the organic Mn proteinate (Wang et al., 2012) did not markedly affect the performance of broilers. Thus, new organic Mn chelates, which not only increase the absorption in the intestine but also improve the growth performance, are needed for broilers.

dl-2-hydroxy-4-(methylthio) butanoic acid (MHA) improved serum antioxidant capacity (Yang et al., 2016), enhanced humoral and nonspecific immune competence (Zhang and Guo, 2008), and improved growth performance in broilers (Willemsen et al., 2011; Zou et al., 2015). Mn methionine hydroxyl analog chelated (Mn-MHAC) is a newly designed Mn fortifier, in which the effects on broilers are poorly known. Mn-methionine chelates levels improved the ADFI, ADG, gain-to-feed ratio, and incidence of leg abnormality of broilers (Li et al., 2005). However, its effects and mechanisms as compared with the inorganic Mn sources on those parameters need further investigation.

Therefore, this study aimed to evaluate the effects of Mn-MHAC on growth performance, trace element deposition, and antioxidant activity in Arbor Acres commercial broilers, and to recommend the proper Mn-MHAC level to meet the Mn requirement of broilers.

## 2 Materials and methods

### 2.1 Ethics statement

Animal experiments were approved by the Animal Care Committee of the Institute of Subtropical Agriculture, Chinese Academy of Science (2015-8A).

### 2.2 Birds and management

Four hundred and thirty-two 10-d-old Arbor Acres commercial female broilers with similar body weight were selected from a commercial flock (Changsha County, Changsha City, China). The experimental chickens were then divided into 6 equal groups (6 replicates/group, 12 birds/replicate). Four birds were housed in a (65 cm × 50 cm × 35 cm) wire cage with three ladders, and 3-wire cages formed an experimental unit that was randomly distributed in the shed. The lighting regimen used was a 16 h light and 8 h darkness cycle with lights beginning at 06:00 local time, July to August. All birds were allowed ad libitum access to water throughout the experiment period. The trial lasted for 5 weeks, from d 10 to 45 of the age of the broilers.

### 2.3 Diets

The basal corn-soybean meal diets (Table 1) were formulated to meet or exceed the requirements for Arbor Acres commercial broilers (NRC, 1994) except for Mn. The broilers were fed the basal diet containing 25.64 mg Mn/kg diet for 10 d, after which they were supplemented with Mn at 0, 100 mg/kg from Mn sulfate and at 25, 50, 75 and 100 mg/kg from Mn-MHAC, respectively. The levels of Mn and MHA in Mn-MHAC were 10.24% and 33%, respectively (Xing-Jia bio-engineering Co., Ltd., Changsha, China). All broilers were fed corn-soybean-based diets in mash form. The analyzed Mn concentration in basal diet and feeds supplemented with Mn sulfate (100 mg Mn/kg diet) and Mn-MHAC (25, 50, 75, and 100 mg Mn/kg diet) are presented in Table 2.

**Table 1:** The ingredients and nutrient level of the basal diet for the broilers (dry matter basis).

Item	Content
Ingredients, %	
Corn	60.50
Soybean meal (46%)	28.00
Wheat bran	5.50
Soybean oil	2.00
Dicalcium phosphate	1.30
NaCl	0.32
Sodium bicarbonate	0.20
Limestone meal	1.50
dl-Methionine	0.17
Premix^1^	0.51
Total	100.00
Nutrient level^2^, g/kg	
ME, kcal/kg	3,032.70
Crude protein	199.63
Ca, %	1.04
Total P, %	0.69
Available P, %	0.45
Methionine	4.57
Methionine + Cystine	7.89
Lysine	10.03
Threonine	6.88
Valine	9.03
Isoleucine	7.49
Tryptophan	2.22
Mn, mg/kg	25.64

1 Supplied per kilogram of diet: 12,000 IU of vitamin A; 3,000 IU of vitamin D_3_; 30 mg of vitamin E; 6 mg of vitamin K_3_; 3 mg of vitamin B_1_; 9 mg of vitamin B_2_; 6 mg of vitamin B_6_; 0.03 mg of vitamin B_12;_ 0.15 mg of d-biotin; 18 mg of d-pantothenic acid; 1.5 mg of folic acid; 6 mg of nicotinamide; 18.15 mg of ethoxyquin; 50 mg of choline chloride; 10 mg of phytase; 0.004 mg of ubiquitin Ca; 5.12 mg of Cu; 72 mg of Fe; 56 mg of Zn; 0.64 mg of I; 0.24 mg of Se; 0.32 mg of Co.

2 All of the values are calculated except that of Mn was analysed.

**Table 2:** Analyzed manganese (Mn) concentrations in experimental diets (mg/kg).

Item	Mn level	
	Supplemental	Final total
Basal diet	0	25.64
Basal diet + Mn sulfate	100	115.32
Basal diet + Mn-MHAC	25	55.53
Basal diet + Mn-MHAC	50	76.95
Basal diet + Mn-MHAC	75	102.94
Basal diet + Mn-MHAC	100	124.56

Mn-MHAC = manganese methionine hydroxy analog chelated.

### 2.4 Sample collection and analytical determination

#### 2.4.1 Sample collection

At 46 d of age, 3 mL of blood was collected from the jugular vein of one bird from each replicate and was kept in sterilized glass vacuum containers at 4 °C for 2 h. The collected blood samples were centrifuged at 2,500 × *g* for 5 min in a temperature-controlled centrifuge (4 °C; Thermo Scientific Bench top Centrifuge). Then the serum was stored at −20 °C in polyethylene tubes for further mineral analysis and Mn superoxide dismutase (Mn-SOD) and copper-zinc superoxide dismutase (Cu/Zn-SOD) activities analysis. Broilers were killed by cervical dislocation. The tibia and about 10 g of the liver and heart were removed and frozen at −20 °C for testing Cu, Fe, and Mn contents. Approximately 2 g of the heart was collected for Mn-SOD and Cu/Zn-SOD enzymatic activity assay. About 2 cm of the duodenum, jejunum, and ileum were removed and immediately frozen in liquid nitrogen for mRNA expression analysis of divalent metal transporter 1 *(DMT1*), transferrin (*TF*), transferrin receptor (*TFRC*), ferroportin 1 (*FPN1*), cutC copper transporter (*CUTC*), high-affinity Cu transporter-1 (*CTR1*) and low-affinity Cu transporter-2 (*CTR2*).

#### 2.4.2 Growth performance

All of the broilers were weighed at the start and the end of the experiment to calculate the ADG. The ADFI and mortality were also recorded during the experiment. The feed-to-gain ratio (F:G) is expressed as F:G = ADFI/ADG.

#### 2.4.3 Measure of enzyme activity in serum and heart

The Mn superoxide dismutase (Mn-SOD) and copper-zinc superoxide dismutase (Cu/Zn-SOD) activities in serum and the heart were determined according to the manufacturer instructions of kits (Nanjing Jiancheng Biotechnology Institute, China) (Meng et al., 2019).

#### 2.4.4 Trace elements analyses

The concentrations of Fe, Cu and Mn in the feed, serum, liver and heart of broilers were determined with an inductively coupled plasma emission spectrometer (ICP) as described by Wan et al. (2018). Briefly, the tissue samples (2.00 ± 0.05 g), feed (1.00 ± 0.05 g), and serum (2.00 ± 0.05 mL) were weighed in triplicate and subjected to acid digestion using mixture acids (HNO_3_: HCLO_4_ = 4:1) 15 mL following a heating procedure: 80 °C, 60 min; 120 °C, 30 min; and 180 °C, 30 min. These samples were dried at 260 °C and re-dissolved with 5 mL of 1% HNO_3_. Then they were transferred to a 25-mL volumetric flask and diluted with 1% HNO_3_ before being submitted for ICP analyses (ICP-720ES, Agilent, Palo Alto, CA, USA). Trace element in the tibia were determined by ICP, as described by Lin et al. (2018).

#### 2.4.5 RNA extraction, reverse transcription and real-time PCR analysis

To quantify the mRNA expression level, the total RNA was isolated from the small intestinal tissue (approximately 50 mg) using TRIzol reagent (Beyotime Biotechnology, Shanghai, China) according to manufacturer instructions. The first-strand cDNA was then synthesized, and meanwhile, genomic DNA was removed using a reverse transcription kit (Takara Biomedical Technology, Japan). All primers were designed in NCBI using the chick gene sequence to produce an amplification product (Table 3). Real-time PCR was then performed as previously described (Liu et al., 2019). The relative mRNA expression level was calculated using the 2^−ΔΔCt^ method after normalization with β-actin as a housekeeping gene.

**Table 3:** Sequence of primers for real-time PCR.

Target gene	Accession no.	Nucleotide sequence of primers (5′ to 3′)	Product size, bp
*DMT1*	NM_001128101.1	F: AGCCGTTCACCACTTATTTCG R: GGTCCAAATAGGCGATGCTC	129
*TF*	NM_205304.1	F: GCAGTAGGCAAGGATGAGAAGAG R: CCACAGACACCAGCAGTATAGAC	173
*TFRC*	NM_205256.2	F: GGTGCTACTGAATGGCTGGAG R: CTCTGAGACTGCTGCTGGATTC	180
*FPN1*	NM_001012913.1	F: CCAGACCTCCTTGGTTGTTCAG R: AGCACATCGTAAGAAGCCATCC	122
*CUTC*	NM_001006503.1	F: GCGATAGCTCTGCACTGGAAG R: CCTGGCACTACGACAATTCTACC	82
*CTR1*	NM_001305660.1	F: ACCACGCATCCTGAACATCAC R: AGAACATGGCTAGGAAGAAGACAG	190
*CTR2*	NM_001277685.1	F: GGTGGTTCCGGTACCATGTG R: TAAGACATGACAGCCAGCATCAG	88
β-actin	NM_205518.1	F: TTACTCGCCTCTGTGAAGGC R: TCCTAGACTGTGGGGGACTG	228

*DMT1* = divalent metal transporter 1; *TF* = transferrin; *TFRC* = transferrin receptor; *FPN1* = ferroportin 1; *CUTC* = cutC copper transporter; *CTR1* = Cu transporter-1; *CTR2* = Cu transporter-2; F = forward; R = reverse.

### 2.5 Statistical analysis

All statistical analyses were performed using SPSS 17.0 software and shown as means ± standard error of the mean (SEM). Significant differences among different treatment groups were evaluated by one-way analysis of variance (ANOVA) followed by Duncan's multiple-ranges test. Then, the linear and quadratic effects of Mn-MHAC levels were analyzed using regression analysis in SPSS 17.0. Probability values < 0.05 were considered statistically significant.

## 3 Results

### 3.1 Growth performance

The growth performance of broilers is presented in Table 4. Compared with the control group, dietary supplementation with Mn sulfate (100 mg Mn/kg diet) and Mn-MHAC (50, 75, and 100 mg Mn/kg diet) significantly increased the BW and ADG of broilers (*P* < 0.001). Notably, the broilers supplemented with Mn-MHAC (50 mg Mn/kg diet) had significantly greater BW, ADFI, and ADG compared with the other Mn-MHAC groups (*P* < 0.05). The relationships between dietary Mn-MHAC levels and BW or ADG were described by a quadratic model (*P* = 0.017 and 0.017, respectively). No significant differences were observed among treatment groups in the F:G and the mortality rate of the broilers for the entire feeding trial (*P* > 0.05).

**Table 4:** Effects of Mn-MHAC on growth performance from d 10 to 45.^1^

Item	Dietary treatment						SEM	*P*-value	Polynomial contrasts, *P*-value	
	Control (0 mg Mn/kg)	Mn sulfate (100 mg Mn/kg)	Mn-MHAC (25 mg Mn/kg)	Mn-MHAC (50 mg Mn/kg)	Mn-MHAC (75 mg Mn/kg)	Mn-MHAC (100 mg Mn/kg)			Mn-MHAC linear	Mn-MHAC quadratic
BW^2^, kg	1.86^c^	2.02^ab^	1.94^bc^	2.07^a^	1.99^b^	1.96^b^	0.015	<0.001	0.940	0.017
ADFI^3^, g	88.04^b^	94.09^b^	91.13^b^	100.44^a^	89.99^b^	92.47^b^	0.982	0.002	0.926	0.114
ADG^4^, g	47.75^c^	52.17^ab^	49.87^bc^	53.91^a^	51.40^b^	50.50^b^	0.452	<0.001	0.940	0.017
F:G, g/g	1.85	1.80	1.83	1.80	1.79	1.83	0.015	0.308	0.994	0.186
Mortality	6.94	5.56	9.72	6.94	11.11	5.56	1.344	0.815	0.653	0.876

Mn-MHAC = manganese methionine hydroxy analog chelated; BW = body weight; ADG = average daily gain; ADFI = average daily feed intake; F:G = feed-to-gain ratio.

^a, b, c^ Values in the same row with different superscript are significantly different (*P* < 0.05) by one-way ANOVA.

1 Data are means of 6 replicates of 12 broilers per dietary treatment.

2 BW Regression equation: *y* = −4.880*x*^2^ + 0.006*x* + 1.856, *R*^2^ = 0.441.

3 ADFI Regression equation: *y* = −0.002*x*^2^ + 0.271*x* + 87.874, *R*^2^ = 0.193.

4 ADG Regression equation: *y* = −0.001*x*^2^ + 0.172*x* + 47.485, *R*^2^ = 0.441.

### 3.2 Mn, Cu and Fe depositions in serum

Serum Mn concentration significantly increased when broilers were supplemented with Mn-MHAC (50 or 100 mg Mn/kg diet) and Mn sulfate (100 mg Mn/kg diet) (*P* = 0.042). Similarly, serum Cu concentration increased (*P* < 0.001) markedly in Mn-MHAC groups (50, 75, or 100 mg Mn/kg diet) compared with both the control and Mn sulfate groups. No significant difference was observed in the serum Fe concentration among experimental groups (*P* > 0.05) (Table 5).

**Table 5:** Effects of Mn-MHAC on trace element deposition.^1^

Item, μg/g	Dietary treatment						SEM	*P*-value	Polynomial contrasts, *P*-value	
	Control (0 mg Mn/kg)	Mn sulfate (100 mg Mn/kg)	Mn-MHAC (25 mg Mn/kg)	Mn-MHAC (50 mg Mn/kg)	Mn-MHAC (75 mg Mn/kg)	Mn-MHAC (100 mg Mn/kg)			Mn-MHAC linear	Mn-MHAC quadratic
Serum										
Mn^2^	0.103^b^	0.177^a^	0.145^ab^	0.156^a^	0.113^b^	0.172^a^	0.0086	0.042	0.963	0.657
Cu^3^	0.212^c^	0.203^c^	0.359^bc^	0.800^a^	0.601^ab^	0.614^ab^	0.0516	<0.001	0.368	0.161
Fe	3.975	3.321	4.414	4.364	3.118	4.46	0.2387	0.497	0.811	0.722
Liver										
Mn	2.676	2.843	2.677	2.859	3.068	2.939	0.0672	0.584	0.342	0.363
Cu^4^	4.075	3.975	4.693	4.758	4.615	4.863	0.1111	0.064	0.661	0.782
Fe	279.582	271.446	245.443	220.733	265.490	289.364	12.5936	0.639	0.160	0.377
Heart										
Mn	0.465	0.418	0.446	0.423	0.412	0.436	0.0111	0.753	0.865	0.639
Cu	3.181	3.005	3.063	3.034	3.278	3.118	0.0479	0.610	0.539	0.742
Fe^5^	35.49	29.887	32.914	33.480	29.929	27.262	1.2923	0.120	0.020	0.052
Tibia										
Mn^6^	1.789^b^	2.127^b^	1.992^b^	2.179^b^	3.046^a^	2.990^a^	0.1141	<0.001	0.002	0.009
Cu	2.077	2.140	2.262	2.043	2.154	2.587	0.0973	0.637	0.249	0.170
Fe^7^	70.757^a^	54.914^b^	60.633^ab^	57.229^b^	53.814^b^	55.200^b^	1.7235	0.029	0.286	0.475

Mn-MHAC = manganese methionine hydroxy analog chelated; Mn = manganese; Fe = iron; Cu = copper.

^a, b, c^ Values in the same row with different superscript are significantly different (*P* < 0.05) by one-way ANOVA.

1 Data are means of 6 replicates of 12 broilers per dietary treatment.

2 Serum Mn Regression equation: *y* = 0.009*x* + 0.101, *R*^2^ = 0.103.

3 Serum Cu Regression equation: *y* = −0.028*x*^2^ + 0.286*x*-0.072, *R*^2^ = 0.305, *P* = 0.004 or *y* = 0.093*x* + 0.169, *R*^2^ = 0.249.

4 Liver Cu Regression equation: *y* = 0.006*x* + 4.240, *R*^2^ = 0.125.

5 Heart Fe Regression equation: *y* = 0.001*x*^2^ - 0.146*x* + 38.855, *R*^2^ = 0.292.

6 Tibia Mn Regression equation: *y* = 1.772*x*^2^ + 0.012*x* + 1.733, *R*^2^ = 0.419.

7 Tibia Fe Regression equation: *y* = 0.003*x*^2^ - 0.414*x* + 70.441, *R*^2^ = 0.282.

### 3.3 Mn, Cu and Fe depositions in liver and heart

Heart Fe decreased linearly with the addition of Mn-MHAC (*P* = 0.020) (Table 5). There were no significant differences in hepatic Mn and Fe concentrations, as well as heart Mn and Fe concentrations among experimental groups (*P* > 0.05).

### 3.4 Mn, Cu and Fe depositions in tibia

The tibial Mn level was significantly higher in the Mn-MHAC groups that were supplemented with the 75 or 100 mg Mn/kg diet, compared with the other groups (*P* < 0.001), and the relationship with dietary Mn-MHAC levels was described by a linear or quadratic model (*P* = 0.002 and 0.009, respectively). Tibial Fe concentration markedly decreased in the Mn-MHAC groups that were supplemented with 50, 75, 100 mg Mn/kg diet or Mn sulfate group when compared with the control group (*P* = 0.029). The concentration of Cu in tibia was not significantly affected by dietary Mn levels (*P* > 0.05) (Table 5).

### 3.5 Mn-SOD and Cu/Zn-SOD activities in serum and heart

As shown in Table 6, the Mn-MHAC and Mn sulfate supplementation markedly lowered serum Mn-SOD activity (*P* < 0.001), and serum Mn-SOD activity decreased with the Mn-MHAC level (linear or quadratic, *P* = 0.048 and 0.019, respectively). No significant differences were found in Mn-SOD and Cu/Zn-SOD activities in the heart and Cu/Zn-SOD activities in serum among different groups (*P* > 0.05).

**Table 6:** Effects of Mn-MHAC on Mn-SOD and Cu/Zn-SOD activities in the serum and heat.^1^

Item	Dietary treatment						SEM	*P*-value	Polynomial contrasts, *P*-value	
	Control (0 mg Mn/kg)	Mn sulfate (100 mg Mn/kg)	Mn-MHAC (25 mg Mn/kg)	Mn-MHAC (50 mg Mn/kg)	Mn-MHAC (75 mg Mn/kg)	Mn-MHAC (100 mg Mn/kg)			Mn-MHAC linear	Mn-MHAC quadratic
Serum										
Mn-SOD^2^, U/mL	134.51^a^	91.95^b^	83.45^bc^	65.74^cd^	58.93^d^	66.12^cd^	5.071	<0.001	0.048	0.019
Cu/Zn-SOD, U/mL	73.58	59.78	74.04	81.99	89.47	96.92	6.777	0.724	0.315	0.610
Heart										
Mn-SOD, U/mL	155.18	151.35	160.34	169.80	194.54	160.54	8.011	0.698	0.441	0.561
Cu/Zn-SOD, U/mL	186.68	267.15	177.05	169.74	170.03	161.84	8.284	0.001	0.654	0.896

Mn-MHAC = manganese methionine hydroxy analog chelated; Mn-SOD = Mn superoxide dismutase; Cu/Zn-SOD = copper-zinc superoxide dismutase.

^a, b, c, d^ Values in the same row with different superscript are significantly different (*P* < 0.05) by one-way ANOVA.

1 Data are means of 6 replicates of 12 broilers per dietary treatment.

2 Serum Mn-SOD Regression equation: *y* = −1.055*x*^2^ - 0.017*x* + 5.981, *R*^2^ = 0.548.

### 3.6 The relative mRNA levels of Cu, Fe or Mn transporters in the small intestine of broilers

Based on the above results, the relative mRNA levels of *DMT1*, *TF*, *TFRC*, *FPN1*, *CUTC*, *CTR1*, and *CTR2* were investigated. As shown in Table 7, the relative mRNA levels of *DMT1* (*P* = 0.024), *FPN1* (*P* = 0.049), and *CTR1* (*P* < 0.001) in the duodenum, as well as the relative mRNA levels of *CTR1* in the jejunum and ileum (*P* = 0.040 and 0.011, respectively), were higher in the Mn-supplemented group than the control group. The Mn sulfate supplementation up-regulated the mRNA expression level of *DMT1* in the jejunum and ileum (*P* = 0.023 and 0.047, respectively) compared with both the control and the Mn-MHAC groups.

**Table 7:** The relative mRNA levels of Cu, Fe or Mn transporter genes in the small intestine of broilers.^1^

Item	Dietary treatment			SEM	*P*-value
	Control (0 mg Mn/kg)	Mn sulfate (100 mg Mn/kg)	Mn-MHAC (50 mg Mn/kg)		
Duodenum					
*TFRC*	1.00	1.35	1.18	0.131	0.580
*TF*	1.00	1.22	0.97	0.099	0.563
*FPN1*	1.00^b^	2.05^a^	1.83^a^	0.190	0.049
*DMT1*	1.00^b^	1.52^a^	1.62^a^	0.106	0.024
*CUTC*	1.00	1.26	0.96	0.091	0.362
*CTR2*	1.00	1.15	0.96	0.080	0.610
*CTR1*	1.00^b^	3.70^a^	2.69^a^	0.337	<0.001
Jejunum					
*TFRC*	1.00	1.01	0.84	0.075	0.594
*TF*	1.00	0.94	0.95	0.066	0.934
*FPN1*	1.00	1.09	0.75	0.072	0.140
*DMT1*	1.00^b^	1.36^a^	0.94^b^	0.073	0.023
*CUTC*	1.00	1.17	0.88	0.070	0.238
*CTR2*	1.00	1.14	1.21	0.078	0.569
*CTR1*	1.00^b^	2.37^a^	2.45^a^	0.265	0.040
Ileum					
*TFRC*	1.00	0.99	0.98	0.064	0.992
*TF*	1.00	0.91	1.10	0.083	0.685
*FPN1*	1.00	1.22	1.10	0.104	0.709
*DMT1*	1.00^b^	1.60^a^	1.10^b^	0.110	0.047
*CUTC*	1.00	1.51	1.22	0.114	0.211
*CTR2*	1.00	0.84	0.86	0.053	0.412
*CTR1*	1.00^b^	1.47^a^	1.63^a^	0.101	0.011

Cu = copper; Fe = iron; Mn = manganese; Mn-MHAC = manganese methionine hydroxy analog chelated; *TFRC* = transferrin receptor; *TF* = transferrin; *FPN1* = ferroportin 1; *DMT1* = divalent metal transporter 1; *CUTC* = cutC copper transporter; *CTR2* = Cu transporter-2; *CTR1* = Cu transporter-1.

^a, b^ Values in the same row with different superscript are significantly different (*P* < 0.05) by one-way ANOVA.

1 Data are means of 6 replicates of 12 broilers per dietary treatment.

## 4 Discussion

Manganese is a micronutrient critical for chicken survival and development. It has been reported that Mn deficiency significantly decreased growth rate and feed intake compared to basal diets (Bolze et al., 1985). In the present study, the results showed that either organic or inorganic Mn supplementation improved the growth performance of broilers. Similarly, previous studies reported that organic Mn supplementation decreased the F:G in broilers (Bao et al., 2007; 2010). Furthermore, the present study showed that the broilers supplemented with Mn-MHAC sources (50 mg Mn/kg diet) had optimal BW gain and were 11.3% heavier than the birds fed the basal diet. In this regard, our results suggest that Mn-MHAC had a similar effect to the organic Mn that was reported by Bao (Bao et al., 2010), and is more effective than Mn fumarate (Berta et al., 2004) or organic Mn proteinate (Wang et al., 2012). It was reported that the biological effects of organic Mn chelates are higher than the Mn sulfate, which suggests that dietary supplementation with organic Mn chelates is feasible for improving the growth performance of broilers (Henry et al., 1989). Notably, the results of the current study demonstrate that the effects of supplementation with Mn-MHAC source (25, 50 or 75 mg Mn/kg diet) were as efficacious as Mn sulfate (100 mg Mn/kg diet) on growth performance of broilers, indicating that the inorganic Mn could be replaced by the lower concentration of Mn-MHAC. The improved growth of broilers that were supplemented with Mn-MHAC could have been explained partly by the increased feed intake.

Mn-SOD is the primary antioxidant enzyme in the mitochondria that plays a critical role in the detoxification of superoxide free radicals and protects cells from oxidative stress (Das et al., 1997). Moreover, Mn-SOD is an essential Mn-containing enzyme (Li and Zhou, 2011). Previous studies showed that heart Mn-SOD activity increased when chickens were supplemented with 60 to 300 mg Mn/kg in their diet (Bai et al., 2014; Li et al., 2004). In comparison, Luo et al. (2007) reported that heart Mn-SOD activity lacked the sensitivity required to detect differences among Mn sources, which is consistent with our results. In this study, we found that serum Mn-SOD activity decreased significantly when chickens were fed the Mn-MHAC supplemented diet. This could be associated with the serum Mn concentration, which has an increased tendency with the dietary Mn-MHAC levels to rise on the whole. The heart Cu/Zn-SOD activity increased when broilers were fed the 300 mg Mn/kg diet (Bai et al., 2014), whereas the present study showed that Cu/Zn-SOD activities in the heart and serum showed no significant difference when broilers were fed Mn at 100 mg/kg. Therefore, the improved growth performance could be attributed to high Mn and Cu concentrations in the serum of broilers fed a diet supplemented with Mn-MHAC.

Mineral accumulation in the tissue of broilers was considered a sensitive criterion for mineral utilization. Tibial Mn, which increased linearly with increasing dietary Mn levels, was an excellent indicator for assessing relative Mn bioavailability (Henry et al., 1989; Yan and Waldroup, 2006). Consistently, our study showed that tibial Mn concentrations were linear and increased quadratically in the Mn-MHAC groups, but the heart and hepatic Mn accumulation in chickens were not sensitive enough to detect organic Mn supplementation. A similar result was also found by Berta et al. (2004), who demonstrated that the tibia showed the highest response to Mn, followed by the liver and kidney to a lesser extent, then the heart. The result of serum Mn elevated markedly in Mn-supplemented groups, regardless of dietary sources of Mn, indicating that Mn sources could increase the Mn status of the body.

It has been reported that divalent Fe, Cu, and Mn were competitively or synergistically absorbed (Bao et al., 2010; Fischer Walker et al., 2005). Excessive Mn in the diet may interfere with the metabolism of Fe and Cu in tissues (Soetan, 2010; Shao et al., 2012). The duodenal and hepatic Fe decreased as dietary Mn increased from 60 to 300 mg/kg diet (Bai et al., 2014). Similarly, Fe deposition in the pancreas and liver decreased with dietary Mn supplementation at 3,000 or 1,000 mg/kg diet (Baker and Halpin, 1991; Black et al., 1985). Consistently, in the present study, Fe deposition in tibia and heart decreased with dietary Mn level in feeds. Interestingly, Bao et al. (2010) showed that decreased tibial Fe tended to increase tibia bone strength. Iron is a microelement that is essential for various physiological functions, including electron transfer, oxygen transport and storage, energy metabolism, and redox reactions (Farina et al., 2013). The Fe stores in tissues decreased in proportion to the dietary Mn levels because Mn and Fe share common binding sites in the gut mucosa, and both are transported by transferrin in the blood (Bai et al., 2014). Thereby, an antagonistic relationship between dietary Fe and Mn in absorption and metabolism existed. In the present study, the increased Cu concentrations in serum in Mn-MHAC groups suggested that organic Mn supplementation markedly increased intestinal Cu absorption and enhanced Cu status in birds. A similar result showed that Cu concentration in the liver of lambs increased when fed a diet supplemented with organic Mn (Gresakova et al., 2016). Similarly, Shao and his colleagues showed that the heart Cu increased in a dose and time-dependent manner when supplemented with organic Mn, indicating a synergistic relationship between dietary Cu and Mn in absorption and metabolism (Shao et al., 2012). As an essential micronutrient, Cu is vital for many Cu-dependent processes, including mitochondrial oxidative phosphorylation, free radical detoxification, pigmentation, neurotransmitter synthesis, and iron metabolism (Festa and Thiele, 2011). Differently, inorganic Mn sources did not seem to influence Cu status compared with the control group.

To further explain the differences of Mn, Cu, and Fe concentrations in the serum, liver, heart, and tibia tissues in the different treatment groups, the mRNA levels of *DMT1*, *TF*, *TFRC*, *FPN1*, *CUTC*, *CTR1*, and *CTR2* were investigated. *DMT1* is a highly conserved uptake transporter that utilizes the proton gradient to drive the uptake of several divalent metals, including Fe, Cd, Co, Cu, Zn, and to a lesser extent Ni and Pb, and it plays a pivotal role in Mn absorption in the intestine (Bai et al., 2008; Wang et al., 2006). Our results showed that the mRNA levels of duodenal *DMT1* in Mn sources increased in the supplemented groups as compared to the control group. A similar result showed that this elevated *DMT1* expression directly related to the effect of an enhanced Mn diet (Garcia et al., 2006). Several reports indicate that organic Mn sources exhibit greater transport and absorption than inorganic Mn, and the DMT1 might enhance the organic Mn transport in broiler intestine (Bai et al., 2012; Ji et al., 2006). However, our results found that Mn sulfate (100 mg Mn/kg diet) up-regulated the mRNA levels of *DMT1* in the jejunum and ileum compared with the control and Mn-MHAC groups. In comparison, Mn-MHAC (50 mg Mn/kg diet) did not affect the mRNA levels of *DMT1* in the jejunum and ileum compared with the control group. The reason for the discrepancy was partly explained by the fact that different absorption mechanisms of inorganic Mn and organic Mn or different intestinal segments were used. *FPN1*, the cytoplasmic Fe exporter, is responsible for the entry of Fe into plasma (Ganz and Nemeth, 2006). Recently, Liao et al. has shown that both the mRNA and protein level of *FPN1* were affected by the intestinal segment of broilers fed different Mn sources, demonstrating that *FPN1* was involved in Mn transport in the small intestine of broilers (Liao et al., 2019). This is in line with our results. Several studies found that Mn efflux is mediated by *FPN1*, and Mn and Fe may also share efflux transport mechanisms (Madejczyk and Ballatori, 2012; Yin et al., 2010). In the present study, the relative mRNA levels of duodenal *FPN1* in Mn-supplemented groups increased compared to the control group, suggesting that supplementation of Mn sources may lead to an increase of Mn or Fe efflux, which is linked to decreased Fe in the tibia or heart. Consistently, a study on human embryonic kidney (HEK293T) cell indicated that Mn exposure promotes *FPN1* protein expression, then reduces net Mn accumulation (Yin et al., 2010). *CTR1* is believed to be the primary protein responsible for the import of dietary Cu across the brush border microvilli (Nose et al., 2006; Ohrvik and Thiele, 2014). Moreover, Cu related gene *CTR1* in the duodenum, jejunum, and ileum of broilers in groups supplemented with Mn sources increased, which could be associated with Cu concentrations in serum and tissues. This result suggests that organic Mn supplementation increased small intestinal Cu absorption and enhanced Cu status in birds.

## 5 Conclusions

In summary, the present study showed that supplementation of broiler diets with Mn-MHAC improved growth performance, and regulated tissue trace element deposition, including Fe and Cu. Moreover, the lower concentration of Mn-MHAC could replace inorganic Mn supplementation.

## Author contributions

**Tiantian Meng**: methodology, data curation, formal analysis, investigation, writing- original draft preparation. **Lumin Gao**: investigation, writing- original draft preparation. **Chunyan Xie**: conceptualization, writing-reviewing and editing, project administration. **Yangkui Xiang**: investigation. **Yiqiang Huang**, **Ya-wei** **Zhang**: visualization, resources. **Xin Wu**: writing-reviewing and editing, supervision, resources, funding acquisition.

## Conflict of interest

We declare that we have no financial and personal relationships with other people or organizations that might inappropriately influence our work, and there is no professional or other personal interest of any nature or kind in any product, service and/or company that could be construed as influencing the content of this paper.
